# Gender differences in the psychophysiological effects induced by VOCs emitted from Japanese cedar (*Cryptomeria japonica*)

**DOI:** 10.1186/s12199-018-0700-9

**Published:** 2018-03-12

**Authors:** Eri Matsubara, Shuichi Kawai

**Affiliations:** 1 Forestry and Forest Products Research Institute, 1 Matsunosato, Tsukuba, Ibaraki 305-8687 Japan; 2 Graduate School of Advanced Integrated Studies in Human Survivability, Kyoto University, 1 Nakaadachi-cho, Yoshida, Sakyo-ku, Kyoto, 606-8306 Japan

**Keywords:** Wood interior walls, *Cryptomeria japonica*, VOCs, Psychophysiological effects

## Abstract

**Background:**

Wood is a valuable material for interiors, and the psychophysiological relaxation effects of volatile organic compounds (VOCs) from wood chips and essential oils have been reported. However, few studies have identified the odors in full-scale wooden environment, and also, differences in gender have not been clarified. In this study, we aimed to confirm the effects of VOCs emitted from interior wood walls in both human male and female participants.

**Methods:**

We used Japanese cedar timber and analyzed VOCs in the experimental rooms with and without Japanese cedar timber by gas chromatography-mass spectrometry (GC-MS). The physiological effects were measured using neuroendocrinological and immunological parameters in saliva. A questionnaire was used to evaluate the subjective responses to each odor in the experimental rooms.

**Results:**

The main compound emitted from Japanese cedar timber was δ-cadinene, and the total volume of VOCs in the wood condition (presence of VOCs emitted from Japanese cedar) was 282.4 (μg/m^3^). Significant differences between genders in salivary parameters were shown that there were decreases of α-amylase in wood condition and increases of cortisol in the control (absence of VOCs) condition in female participants compared to male participants. The results demonstrated that VOCs in the experimental room with Japanese cedar timber tend to suppress the activation of the sympathetic nervous activity and non-VOCs of Japanese cedar in the control room increase cortisol in female participants.

**Conclusions:**

These results suggest that an indoor environment with wood interior materials has the potential to be useful for health management, especially women’s health.

## Background

In recent years, wooden materials have been used in more contexts around the world and have increasingly been used for structural and interior materials for houses and public buildings in Japan [1]. Previous studies investigated whether wooden classrooms were considered more comfortable than reinforced concrete ones [2] and whether a wooden interior in the bedroom had the potential to improve sleep quality and decrease subjective fatigue [3]. Previous studies have been conducted on the habitability of rooms with wooden walls, and the psychophysiological favorite effects of interior wood material through visual and tactile pathways have been reported [4, 5]. Volatile organic compounds (VOCs) from wood chips and essential oils produce relaxant effects on the autonomic nervous and electroencephalogram [6–9]. However, few studies have been performed on the psychophysiological responses to odors in a full-scale wooden environment. Moreover, everybody, irrespective of gender, has the opportunity to spend time in wooden spaces; however, differences in gender has not been clarified.

The Japanese cedar (*Cryptomeria japonica*) is a widespread plant species in Japan and the most common tree in Japanese forests. The strength of cedar timber and composite materials such as laminated wood and plywood have been discussed in many previous studies, and Japanese cedar is being widely used in structural and interior construction materials. The VOCs emitted by Japanese cedar give a species-specific odor, and previous reports have shown they produce psychophysiological relaxation effects on the autonomic nervous and electroencephalogram [7, 8]. Furthermore, VOCs emitted in an experimental room with interior walls constructed from Japanese cedar timber have also shown these psychophysiological effects [10–12].

In the present study, we used an experimental room constructed from Japanese cedar [10] to confirm the psychophysiological effects of VOCs with male and female participants. We used monotonous work as a stress task. The physiological effects were measured using neuroendocrinological and immunological parameters to evaluate the physiological responses of participants during and after performing the task. A subjective assessment of VOCs was used to evaluate the psychological responses. We analyzed VOCs in the experimental rooms with and without Japanese cedar timber.

## Methods

### Specifications of the experimental rooms

We used 40-year-old thinned timbers of Japanese cedar from Oguni (Kumamoto, Japan) as the experimental material. The wood drying and processing methods have been described previously [10, 13]. Experimental rooms at Kyoto University (RC structure) were used. The Japanese cedar timbers were set in the experimental room and were designated as the wood condition. Another similar experimental room without Japanese cedar was the control condition. A partition was erected to prevent the timbers from visually influencing the participants. There was no furniture except our experimental tools: a desk, chair, partition, and wood interior panels (only in the wood condition). Temperature and relative humidity (RH) in both rooms were measured with data loggers (TR-72Ui, T & D Corporation, Nagano, Japan).

### Participants and experimental design

This experimental design was approved by the Kyoto University and was in accordance with the Declaration of Helsinki. In total, 27 healthy university students were recruited (17 males and 10 females; age, 21.9 ± 1.5 years; range, 20–25 years). None of the participants had any physical or mental health abnormalities, and none took prescription drugs or smoked. The purpose and schedule for the experiments were explained, and written informed consent was obtained from all participants prior to initiating the study. Consumption of alcohol or medication was prohibited 1 day before the experiment, and caffeine use was prohibited on the day of the experiment. Table 1 shows the experimental design. Each participant performed the experiment twice at a one-week interval: once in the absence (control condition) and once in the presence (wood condition) of VOCs emitted from the Japanese cedar. The order of experimental conditions was counterbalanced between the participants, and none of the participants knew about the room condition prior to the actual experiment.

**Table 1:** Experimental procedure. The arithmetic test was performed in repeated cycles of 15 min of work and 5 min of rest. Saliva was collected from all the participants at pre-test, rest, and post-test time points. A VAS questionnaire on subjective assessment of the experimental rooms was administered at the end of the experimental period

Period	Pre-work	Rest	Work	Rest	Work	Post-work
Time		5 min	15 min	5 min	15 min	
Meas.	Saliva			Saliva		Saliva Subjective assessment

### Gas chromatography-mass spectrometry

The VOCs were collected in the rooms using a carbon tube (ORBO91T; Sigma-Aldrich, St. Louis, MO, USA) by applying a flow rate of 0.1 L min^− 1^ overnight. The VOCs were sampled at the same conditions of psychophysiological experiment without the participants. The VOCs were then analyzed by a gas chromatography-mass spectrometry (GC-MS) system eluted with acetone (GC-MS-QP2010; Shimadzu Co., Ltd., Kyoto, Japan). The system was equipped with an Ultra ALLOY-5 capillary column (30 m × 0.25 mm i.d., 0.25 μm film thickness; Frontier Laboratories Ltd., Fukushima, Japan). The temperature program was as follows: 50 °C for 3 min, followed by increase of 15 °C/min^−1^ to 150 °C, 4 °C/min^−1^ to 170 °C, and 20 °C/min^−1^ to 250 °C, and holding for 5 min. The other parameters were as follows: injection temperature, 250 °C; ion source temperature, 250 °C; carrier inlet pressure, 100 kPa; He, 1.69 ml min^−1^; and injection volume, 1 μl. We compared the GC-MS data with a mass spectral database library (NIST08) and calculated the concentrations of the target compounds in the sample using a β-caryophyllene standard calibration curve (Sigma-Aldrich Japan Co., Tokyo, Japan).

### Arithmetic stress task

We used the Uchida-Kraepelin (U–K) test [14], a serial addition test in which calculations are performed as quickly and accurately as possible. Each participant was supplied with pre-printed paper containing 15 lines of random, single-digit, horizontally aligned numbers and was instructed to add the numbers on a specified line and to move to a new line every minute. This test was performed in repeated cycles of 15 min of work and 5 min of rest (Table 1).

### Subjective assessment of odor in the experimental rooms

The subjective responses to each odor in the experimental rooms were measured after the arithmetic stress task was completed. We used a visual analog scale (VAS) consisting of an eight-item questionnaire designed to differentiate subjective responses: cannot concentrate/can concentrate, dislike/like, feel warm/feel cold, uncomfortable/comfortable, feel restless/feel calm, artificial/natural, feel cozy/not cozy, and bad odor/good odor.

### Salivary stress parameters assay

The neuroendocrinological and immunological parameters in saliva were used to elucidate the physiological effects in the present study. Saliva was collected from all participants immediately before, during, and after they performed the arithmetic stress task and was stored at − 20 °C until analysis. Salivary α-amylase was measured using a salivary amylase monitor (Nipro Co., Osaka, Japan). Salivary cortisol and secreted immunoglobulin A (sIgA) levels were measured with enzyme immunoassay (EIA) kits (Salmetrics, State College, PA, USA). Salivary chromogranin A (CgA) was also measured with an EIA kit (Phoenix Pharmaceuticals Inc., Burlingame, CA, USA).

### Statistical analysis

All values are expressed as mean ± standard error. The Student’s *t* test was used to compare gender differences in both experimental rooms in the stress task performance and the salivary parameters, and the Mann-Whitney *U* test was used to compare the subjective assessments. A *p* value was shown in the figure, and a *p* value of < 0.05 was considered significant. All statistical analyses were performed using SPSS 17.0J for Windows (SPSS Japan, Tokyo, Japan).

## Results

We investigated the effects of VOCs emitted from Japanese cedar during and after a stress task, and saliva was collected and analyzed for stress markers (Table 1). We analyzed VOCs in both experimental rooms. The participants performed an arithmetic stress task and remained seated and quiet during a rest period after the saliva was collected.

### Constituent analysis of VOCs and room temperature and relative humidity

We analyzed the chemical compounds in the experimental rooms by GC-MS. The main compound detected was δ-cadinene, and other sesquiterpenes were α-cubebene, α-copaene, β-cubebene, β-cedrene, β-caryophyllene, thujopsene, α-humulene, γ-amorphene, and α-muulorene. The total volume of VOCs in the experimental room was 282.4 (μg/m^3^). VOCs were undetectable in the control room. The temperature and relative humidity of the control condition were maintained at 21.7 ± 1.2 °C, 56.9 ± 3.2% and that of the wood condition were maintained at 20.4 ± 1.8 °C, 54.3 ± 4.5% throughout the experiment.

### Arithmetic performance

**Fig. 1:**
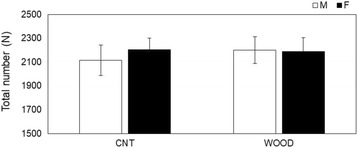
Rate of correct calculations during arithmetic work. Rate of correct calculations during arithmetic work. The bars represent the rate of correct calculations in the control condition (left) and Japanese cedar wood panels (experimental; light) conditions. CNT indicates the control condition, while WOOD indicates the experimental condition: male participants (white) and female participants (black). Differences between both conditions were not significant. Data are shown as mean ± SEM

Performance was determined by the total number of correct calculations made. This was defined as the average of total work time (Fig. 1). No difference in performance was found between gender and the experimental conditions.

### Subjective assessment of odor in the experimental rooms

**Fig. 2:**
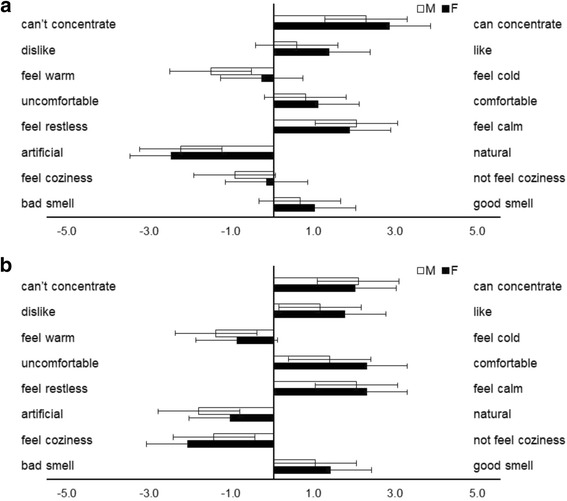
Subjective effects of the odor of the experiment room. The bars in **a** show the visual analog scale scores reported by participants in the control condition: male participants (white) and female participants (black). Differences between conditions were not significant. Data are shown as mean ± SEM. The bars in **b** show visual analog scale scores reported by participants in the Japanese cedar wood panels (experimental) condition; male participants (white) and female participants (black). Differences between conditions were not significant. Data are shown as mean ± SEM

The subjective effects of Japanese cedar were determined using an eight-item questionnaire using a VAS. The VAS was represented by a horizontal line 100 mm long, which was anchored by a word descriptor at each end. The participants marked a point on the line indicating their subjective response to each odor. The VAS score was determined by measuring from the end of the line to the center of the mark in millimeters. The VAS scores tended to be different between gender in the control condition: the “feel warm/feel cold” scores were − 1.5 ± 0.5 for male participants and − 0.3 ± 0.7 for female participants (*p* = 0.084). No gender differences were shown in the wood condition (Fig. 2).

### Salivary stress parameters assay

**Fig. 3:**
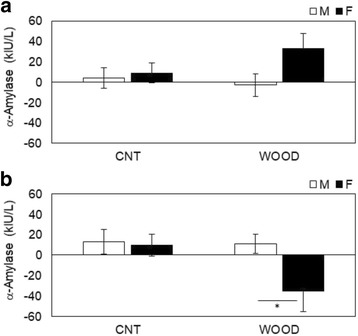
Variation of salivary α-amylase levels changes **a** from during-work level to pre-work and **b** from post-work to during-work. The bar in **a** describes changes in the salivary α-amylase levels of participants in the control condition (left) and Japanese cedar wood panels (experimental; light) conditions: male participants (white) and female participants (black). CNT indicates the control condition, while WOOD indicates the experimental condition. Differences between conditions were not significant. Data are shown as mean ± SEM. The bars in **b** describes changes in the salivary α-amylase levels of participants in the control condition (left) and Japanese cedar wood panels (experimental; light) conditions: male participants (white) and female participants (black). Difference in values between male and female participants was significant (*p* = 0.031). *statistical significance (*p* < 0.05). Data are shown as mean ± SEM

**Fig. 4:**
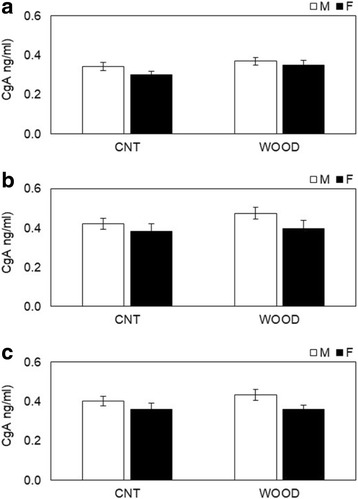
Variation of salivary CgA levels changes **a** pre-, **b** during, and **c** post-experiment measurements. The bar in **a**–**c** describes changes in the salivary CgA levels of participants in the control condition (left) and Japanese cedar wood panels (experimental; light) conditions: male participants (white) and female participants (black). CNT indicates the control condition, while WOOD indicates the experimental condition. Differences between conditions were not significant. Data are shown as mean ± SEM

**Fig. 5:**
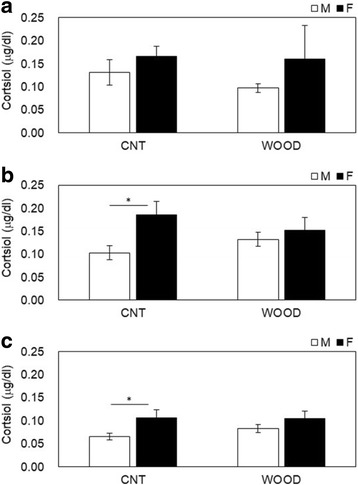
Variation of salivary cortisol levels changes at **a** pre-, **b** during, and **c** post-experiment measurements. The bars in **a**–**c** show changes in the salivary cortisol levels of participants in the control condition (left) and Japanese cedar wood panels (experimental; light) conditions: male participants (white) and female participants (black). CNT indicates the control condition, while WOOD indicates the experimental condition. The bars in **a** show that differences between conditions were not significant. The bars in **b** show that the difference in values between male and female participants were significant (*p* = 0.013) in the control condition. The bars in **c** show that the difference in values between the male and female participants was significant (*p* = 0.027) in the control condition. *statistical significance (*p* < 0.05). Data are shown as mean ± SEM

**Fig. 6:**
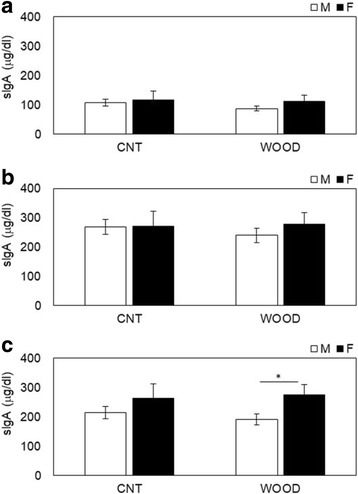
Variation of salivary sIgA levels changes at **a** pre-, **b** during, and **c** post-experiment measurements. The bar in **a**–**c** show the changes in the salivary cortisol levels of participants in the control condition (left) and Japanese cedar wood panels (experimental; light) conditions: male participants (white) and female participants (black).CNT indicates the control condition, while WOOD indicates the experimental condition. The bars in **a** show that the differences between conditions were not significant. The bars in **b** show that the differences between conditions were not significant. The bars in **c** show that the difference in values between male and female participants was significant (*p* = 0.029) in the Japanese cedar wood panels condition. *statistical significance (*p* < 0.05). Data are shown as mean ± SEM

Salivary neuroendocrinological and immunological parameters were measured before, during, and after the participants performed the arithmetic stress task (Table 1). The salivary α-amylase levels changed from the during-work level to those measured pre-work (Fig. 3a) and from post-work to during-work (Fig. 3b). The change in α-amylase levels between during-work and pre-work were − 3.0 ± 10.8 (kIU/L) in male participants and 32.6 ± 15.0 (kIU/L) in female participants in the wood condition (*p* = 0.061) (Fig. 3a). The change of α-amylase levels between post-work and during-work were 11.0 ± 9.6 (kIU/L) in male participants and − 35.4 ± 20.3 (kIU/L) in female participants in the wood condition (*p* = 0.031) (Fig. 3b). No gender differences were shown in the control condition. The changes in salivary CgA are shown in Fig. 4. The differences between genders post-period in the wood condition were 0.43 ± 0.03 (ng/mL) for male participants and 0.36 ± 0.02 (ng/mL) for female participants (*p* = 0.089) (Fig. 4c); no gender differences were shown in all periods in the control condition and other periods in the wood condition. The changes in salivary cortisol were shown in Fig. 5. The significant differences shown between genders during periods in the control condition were 0.10 ± 0.02 (μg/dL) for male participants and 0.19 ± 0.03 (μg/dL) for female participants (*p* = 0.013) (Fig. 5b). The significant differences between genders during periods in the control condition were 0.06 ± 0.01 (μg/dL) for male participants and 0.11 ± 0.02 (μg/dL) for female participants (*p* = 0.027) (Fig. 5c). No gender differences were shown in other periods in the control condition and all periods in the wood condition. The changes in salivary sIgA are shown in Fig. 6. The significant differences between genders post-period in the wood condition were 190.1 ± 18.5 (μg/dL) for male participants and 274.4 ± 35.4 (μg/dL) for female participants (Fig. 6c). No gender differences were shown in all periods in the control condition and other periods in the wood condition.

## Discussion

In the present study, we confirmed that female participants were more affected by different indoor environments than male participants. VOCs in an experimental room constructed from Japanese cedar tended to suppress the activation of sympathetic nervous activity, and non-VOCs of Japanese cedar in a control condition increased cortisol, one type of stress hormone in female participants. These results indicate that interiors made from wood have the potential to be useful for health management, especially women’s health.

The total volume of VOCs emitted from the Japanese cedar was 282.4 (μg/m^3^) in the wood condition, and the constituents were mainly δ-cadinene and other sesquiterpenes. Previous studies reported the characteristics in indoor air and emission behavior of VOCs emitted from solid wood and wood composites [15–18]. The constituents and total volume of VOCs are important factors for creating a comfortable environment good for human health. In this study, we confirmed that the participants did not experience any unpleasant symptoms such as cough, sneeze, or headache during or after the experiment. Cedrol, which is one of the VOCs in Japanese cedar wood, has a sedative effect via modulation of the activity of the sympathetic nervous system and is suggested to function by sympathetic inhibition and increasing dopamine metabolism [19]. β-Caryophyllene is one of the major VOC components of Japanese cedar wood and is suggested to have a vasodilating action by blocking Ca^2+^ influx [20]. We also believe that the other sesquiterpenes have the potential to influence the human body and mental condition. Several previous studies showed the psychophysiological effects caused by an inhalation of total volume of VOCs from several tens of thousands to hundreds [21–23]. However, studies about optimal VOC conditions for the human body, particularly for the sesquiterpenes emitted from a wood interior, have not been reported. Moreover, chronic effects have been researched by several studies, in which seasonal variation in VOCs and the VOC composition of the different interior materials were investigated [17, 18]. A strategy focused on the relationship between indoor air and human health should be adopted for future research.

In the present study, we assessed the responses of participant’s salivary stress, and subjectively, markers were measured under a mentally stressful condition. Task performance was determined by the total number of correct calculations. No difference in performance was observed between genders in both conditions (Fig. 1), which in part was similar to our results reported previously [10]. Offices with indoor plants have the potential to promote beneficial psychophysiological and cognitive effects in working people [24, 25]. In future studies, we should verify the effects of wooden space where people are more psychologically stressed instead of giving them an authentic stress task.

The subjective effects in the experimental rooms tended to be different on one questionnaire item in the control condition (Fig. 2). The difference of gender in our interior materials and subjective assessments was not shown clearly. The subjective parameters of wood odor have been judged for male and female participants in previous reports [7, 11, 12]; therefore, these results might indicate that VOCs of wood inclusive of Japanese cedar could affect people regardless of gender. However, the perception of odors is influenced by many personal factors, including psychological variables, habituation, and social factors. Furthermore, several studies have indicated that the recognition of odor is often poor in patients with allergic rhinitis [26, 27], which suggested that the participants in our study did not have the above problems. However, we should elucidate the subjective effects of volatiles, especially for those who have nasal allergies, in a future study.

Measuring biomarkers in saliva is useful to evaluate stress and fatigue in a noninvasive, convenient way, and the utility of these markers has been demonstrated [28, 29]. In this study, we evaluated time-course changes in α-amylase, CgA, cortisol, and sIgA. Salivary α-amylase is an enzyme that is released from the salivary glands under the control of the sympathetic nervous system [30, 31]. CgA is an acidic glycoprotein produced by the submandibular glands that is secreted into the saliva or released with catecholamine from the sympathetic nervous system nerve endings [32, 33]. α-Amylase and CgA have been used as markers related to the activation of the sympathetic nervous system under stressful conditions. In this study, we observed significant decreases in α-amylase (Fig. 3b) after the arithmetic stress task in female participants compared to male participants. A change in α-amylase is a parameter of the sympathetic nervous system, and our result indicated that VOCs emitted from Japanese cedar induce physiological relaxation in female participants more than male participants at the same period. VOCs emitted from the Japanese cedar experimental room suppressed sympathetic nervous activity in male participants with higher volume of VOCs than that of the present study [10]. These findings suggest that the physiological relaxation effects the female participants experienced occurred at the lower concentration of wood odor. Toda and Morimoto indicated that fragrances induce a decrease in CgA secretion during recovery from an arithmetic task [34], and we obtained a similar decrease in CgA secretion (Fig. 4c), which supported the physiological relaxation effects of female participants. We also observed significant increases in cortisol (Fig. 5b, c) in the control condition during and after the task in female participants compared to male participants. Cortisol is an adrenocortical hormone and is well known as an important stress hormone in the regulation of homeostasis of our body internal environment. Salivary cortisol have been used in various research areas to measure the participant’s stress state; in general, an increase of cortisol secretion reflects psychophysiological, uncomfortable stress [35–37]. Our results showed that female participants became psychologically stressed during stress tasks compared to male participants only in the control condition. The mental stress task used in the present study is an arithmetic work, and task performance was not found different between genders (Fig. 1). The signals to change the work column every minute and a serial addition test which demands completion as quickly and accurately as possible might be possible, especially to induce stress in female participants. A previous study indicated that female participants showed lower cortisol concentrations compared to male participants [38]. However, cortisol secretion is influenced with type of stress exposure and/or other hormones, and individual psychophysiological states are also influenced [39–41]. We also could not find the difference in gender under the wood condition in the resent study. In a future study, we will increase the total number of participants and consider the effects of VOCs on the cortisol secretion in more detail. We also observed significant increases in sIgA (Fig. 6c) in the wood condition after the task in female participants. sIgA plays a major role in the immune system and is increased by acute academic examination [42]. Ring et al. reported that mental arithmetic induces an increase in sIgA concentration and changes in sIgA are under the control of the sympathetic nervous system [43]. Several academic examinations suggested the possibility of lowing sIgA [44], and inhalation of lavender and rosemary odors induced no significant changes [45]. In the present study, we did not investigate the psychophysiological background of participants, but it seems that female participants might be influenced more strongly by several elements affecting the immune system.

We believe that women experience a specific physiological cycle that strongly influences the secretion of neurological and physiological hormones. The hypothalamus is a basal part of the diencephalon and governs the autonomic nervous system and endocrine hormone secretion. The hypothalamic–pituitary system is necessary to secrete the female hormones estrogen and progesterone and the stress hormone cortisol; these hormones affect each other. Regarding the psychological condition, previous study suggested that the range of emotional fluctuation in women is more drastic than that in men [46]. In future studies, we will consider gender-specific effects of VOCs emitted from Japanese cedar.

This study presents some limitations. First, the sample size is fairly small, and there is a bias in the ratio of male to female participants. We should increase the number of participants and gather more evidence in future studies. Secondly, we used only salivary biomarkers to evaluate the physiological effects of relaxation. Next, we will try to measure physiological responses using rats and should investigate the neurological responses and mechanisms of relaxation of wood in more detail in future studies. Thirdly, the results of this study were produced during a transient experiment in only approximately 1 h and have not yet been directly applied to the long-term effects of VOCs. We should accumulate and evaluate evidence gathered during a longer exposure to wooden interior environments. Fourthly, it is unclear which VOC compounds in Japanese cedar are responsible for its relaxation effect. We should explore the compounds in Japanese cedar wood that contribute to this effect in more detail in future studies.

## Conclusion

The possibilities of the positive effects of wood interior environments on the human body have been suggested in many previous studies. Recently, there has been an increasing number of studies to actually measure the psychophysiological condition and vary the effects of wood. In the present study, we found that the gender difference of salivary stress parameters in the experimental conditions and VOCs in an experimental room constructed of Japanese cedar tend to suppress the activation of the sympathetic nervous activity. But this study has some limitations. Therefore, in future studies, we should follow up with more participants, especially female and the general population for the generalization of our findings. Moreover, we should investigate the neurological responses and mechanisms of relaxation of wood, the long-term effects of VOCs, and the compounds in Japanese cedar wood that contribute to relaxation effects.

## Abbreviations

CgA: Chromogranin A
EIA: Enzyme immunoassay
GC-MS: Gas chromatography-mass spectrometry
NIST: National Institute of Standards and Technology
RC: Reinforced concrete
RH: Relative humidity
sIgA: Secreted immunoglobulin A
U–K: Uchida-Kraepelin
VAS: Visual analog scale
VOCs: Volatile organic compounds
